# The Micro-RNA Expression Profiles of Autoimmune Arthritis Reveal Novel Biomarkers of the Disease and Therapeutic Response

**DOI:** 10.3390/ijms19082293

**Published:** 2018-08-05

**Authors:** Steven Dudics, Shivaprasad H. Venkatesha, Kamal D. Moudgil

**Affiliations:** 1 Department of Microbiology and Immunology, University of Maryland School of Medicine, Baltimore, MD 21201, USA; sdudics1@gmail.com (S.D.); hvshivaprasad@gmail.com (S.H.V.); 2 Baltimore Veterans Affairs Medical Center, Baltimore, MD 21201, USA; 3 Division of Rheumatology, Department of Medicine, University of Maryland School of Medicine, Baltimore, MD 21201, USA

**Keywords:** adjuvant arthritis, arthritis, biomarkers, celastrol, inflammation, microRNA, miRNA, rat, rheumatoid arthritis, Traditional Chinese medicine, tripterine, triterpenoid

## Abstract

Rheumatoid arthritis (RA) is a chronic autoimmune disease of the joints affecting about 0.3–1% of the population in different countries. About 50–60 percent of RA patients respond to presently used drugs. Moreover, the current biomarkers for RA have inherent limitations. Consequently, there is a need for additional, new biomarkers for monitoring disease activity and responsiveness to therapy of RA patients. We examined the micro-RNA (miRNA) profile of immune (lymphoid) cells of arthritic Lewis rats and arthritic rats treated with celastrol, a natural triterpenoid. Experimental and bioinformatics analyses revealed 8 miRNAs (miR-22, miR-27a, miR-96, miR-142, miR-223, miR-296, miR-298, and miR-451) and their target genes in functional pathways important for RA pathogenesis. Interestingly, 6 of them (miR-22, miR-27a, miR-96, miR-142, miR-223, and miR-296) were further modulated by celastrol treatment. Interestingly, serum levels of miR-142, miR-155, and miR-223 were higher in arthritic versus control rats, whereas miR-212 showed increased expression in celastrol-treated rats compared with arthritic rats or control rats. This is the first study on comprehensive miRNA expression profiling in the adjuvant-induced arthritis (AA) model and it also has revealed new miRNA targets for celastrol in arthritis. We suggest that subsets of the above miRNAs may serve as novel biomarkers of disease activity and therapeutic response in arthritis.

## 1. Introduction

Rheumatoid arthritis (RA) is a debilitating autoimmune disease characterized by chronic inflammation of the joints along with systemic manifestations [1,2,3]. The prevalence of RA varies from 0.3–1.0 percent globally, and it is more common in developed countries than others [4]. RA is a complex disease involving the interplay among multiple mediators and pathways of inflammation and bone damage. The expression of these mediators and their interactions in turn are controlled by a variety of regulators, including micro-RNAs (miRNAs) [5,6,7]. While current therapies offer a diverse choice of drugs for RA patients, only 50–60% of these patients respond to them [3,8]. Additionally, the currently used biomarkers to assess the development and progression of RA, and to monitor the patients’ responsiveness to treatment, have inherent limitations. Rheumatoid factor (RF) and anti-citrullinated protein antibodies (ACPA) are the mainstay of biomarkers for arthritis, with ACPA exhibiting a similar sensitivity but better predictability of the disease course than RF [9,10]. However, RF and ACPA have also been found in other autoimmune diseases, and ACPA positivity may be limited to a subset of RA patients [11,12]. In view of the above, we proposed that certain miRNAs might serve as novel biomarkers to monitor disease activity and therapeutic response in RA.

The miRNAs are short, non-coding RNA sequences that repress gene expression. They exert their function by binding to the 3’ untranslated region (UTR) sequences of the target messenger RNAs (mRNAs), and either initiate their degradation or inhibit their translation [13,14]. The miRNAs have been studied extensively in the cancer field [15,16,17]. It has been shown that miRNAs can be used as biomarkers for certain cancers, such as breast cancer [18]. Interestingly, miRNAs are gaining increasing recognition for their involvement in autoimmune diseases, as well [19,20,21,22,23,24,25,26]. In RA, miR-146a and miR-155 are among the most studied miRNAs [27]. It has been reported that miR-146a is increased in RA in serum, peripheral blood, CD4^+^ T cells, and synovial tissue [5,27,28,29]. However, a reduction in miR-146a in RA fibroblast-like synoviocytes (FLS) has also been reported, implicating this miRNA in anti-inflammatory effects on FLS [6]. This miRNA is also increased in the joint tissue of osteoarthritis patients [30]. Both miR-146a and miR-155 have also been shown to be increased in IL-1β-stimulated human chondrocytes [14]. Similarly, increased levels of miR-155 in RA and its animal models have been reported [7,20,27,31,32]. However, there is a need to determine the role of additional miRNAs in the progression of RA, as well as their utility as biomarkers of disease activity and/or therapeutic response. In this context, we examined the rat adjuvant-induced arthritis (AA) model of human RA [33,34] and also tested the effect on miRNAs of celastrol, a natural triterpenoid that possesses anti-arthritic activity [35]. Celastrol is a pentacyclic triterpenoid (C_29_H_38_O_4_), and it is a bioactive component of plants belonging to the Celastraceae family, such as *Tripterygium wilfordii* and *Celastrus orbiculatus* [36]. The choice of celastrol in this proof-of-concept study was based on our earlier study showing its beneficial effects against AA [35].

AA is a T cell-mediated autoimmune disease, and it has extensively been used to screen potential new drugs, as well as to define the mechanisms underlying RA pathogenesis [33]. Celastrol, derived from a traditional Chinese herb celastrus, has anti-inflammatory and anti-oxidant properties [35,37]. We have previously shown in the AA model that celastrol possesses anti-arthritic properties. These attributes include inhibition of the pro-inflammatory cytokines [35], skewing of the T helper 17 (Th17)/T regulatory (Treg) cell balance towards immune regulation [37], and modulation of bone remodeling in arthritic rats [35]. Accordingly, we hypothesized that celastrol alters specific miRNAs that are involved in the pathogenesis of AA, and that a subset of these miRNAs may serve as biomarkers for disease progression and responsiveness to therapy. Our results described below support this proposition. We observed that 8 specific miRNAs (miR-22, miR-27a, miR-96, miR-142, miR-223, miR-296, miR-298, and miR-451) have the potential to be key regulators of arthritis pathogenesis. Of these, 6 miRNAs (miR-22, miR-27a, miR-96, miR-142, miR-223, and miR-296) were further modulated following celastrol treatment. The testing of sera of control, arthritic, and celastrol-treated rats further validated the utility of some of these miRNAs as circulating biomarkers. We believe that the above-mentioned miRNAs, whether as a set or individually, could be used in conjunction with current biomarkers for improved diagnosis and/or prognosis of arthritis, as well as for monitoring a patient’s responsiveness to therapeutic intervention. To the best of our knowledge, this is the first study describing a comprehensive miRNA expression profile of rats with AA as well as novel miRNAs targeted by celastrol. The latter may also lead to identification of additional therapeutic targets for arthritis.

## 2. Results

**Figure 1:**
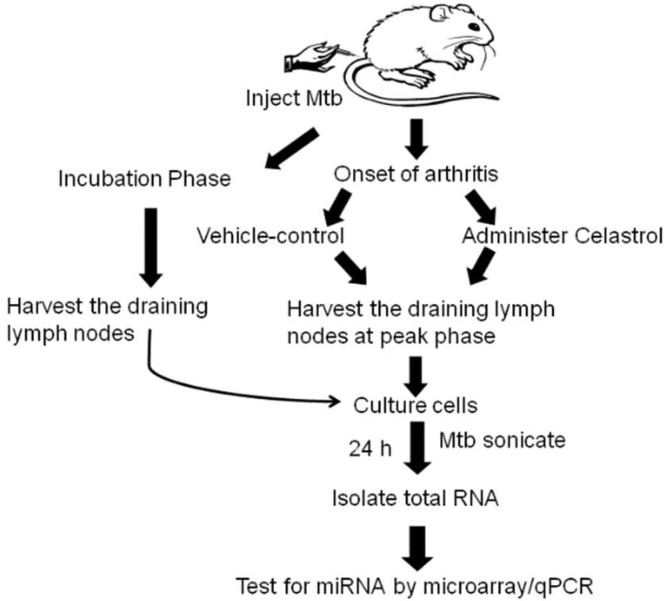
A flow chart showing an overview of the experimental design of the study.

**Figure 2:**
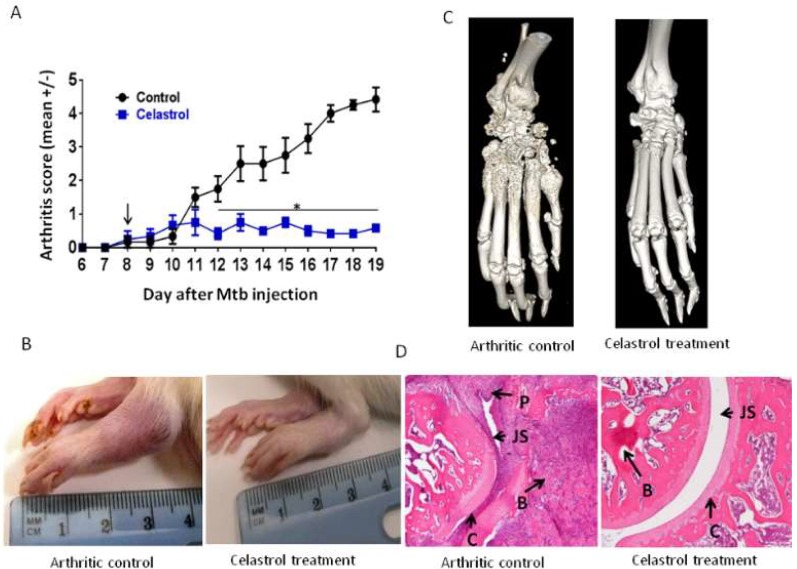
Celastrol inhibits the progression of adjuvant-induced arthritis (AA). (**A**) Mean scores of arthritic Lewis rats (*n* = 4 per group) treated either with celastrol or with the vehicle. Rats were administered celastrol (1 mg/kg) via intraperitoneal (i.p.) injection every day for 3 days starting at the onset of AA, followed by injections every other day until euthanization of rats on day 19 after Mtb injection. (**B**) Photographs, (**C**) computed tomographic (CT) imaging, and (**D**) histological sections of hind paws of vehicle-treated and celastrol-treated rats harvested at peak phase of the disease (day 19). The arrows point to the following: P: pannus; JS: joint space; B: bone; and C: cartilage.

Using the adjuvant arthritis (AA) model in the Lewis rat, we determined the miRNA expression profile of 3 groups of rats: *M. tuberculosis* H_37_R_a_ (Mtb)-immunized rats in Incubation phase of AA, the vehicle-treated arthritic rats, and the celastrol-treated rats) following the experimental design laid out in Figure 1. Rats in the Incubation phase of AA served as the “baseline” control for the other two groups of rats, whereas vehicle-treated rats were compared with celastrol-treated rats (Figure 2). Lymph node cells (LNCs) of these rats were re-stimulated in the presence or absence of antigen (Mtb sonicate) for 24 h. Thereafter, total RNA isolated from these LNCs was tested using miRNA-microarray. As described under Methods, “miRNA elements” represent hybridization intensities in microarray against miRNA probes of rat, mouse, and human origin, as well as multiple probes of one species for a given miRNA. In subsequent analysis, “miRNA” refers to a given RNA sequence represented by the probes of a single species.

### 2.1. LNC Micro-RNA Expression Profile of Incubation Phase Rats and Vehicle-Treated Arthritic Rats

**Figure 3:**
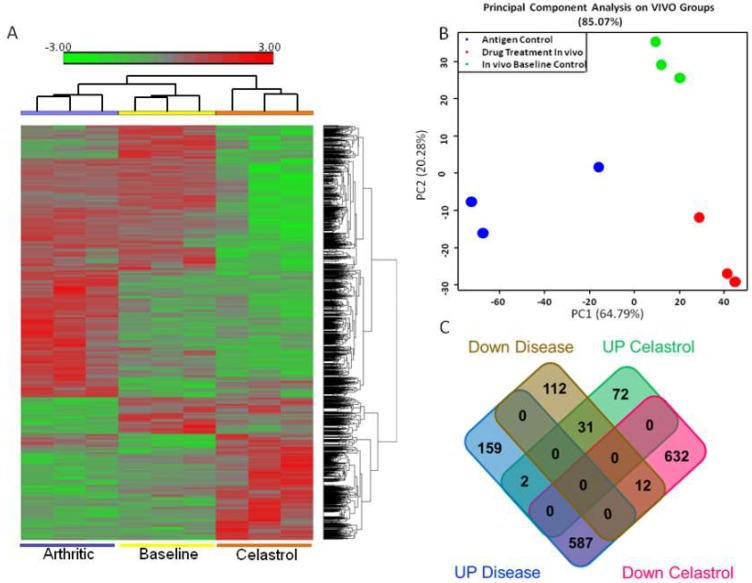
Microarray analysis of the miRNA expression profile of the Incubation phase (baseline) rats, control (vehicle-treated) arthritic rats, and celastrol-treated arthritic rats. (**A**) Heat map of miRNAs expressed in lymph node cells (LNCs) of the above 3 groups of rats (*n* = 3 per group) as indicated in the figure. LNCs were isolated from the draining lymph nodes of rats in the incubation phase of the disease (on day 5 after Mtb injection) and the vehicle-treated or celastrol-treated rats at peak phase of the disease (on day 19 post-Mtb immunization). Thereafter, LNCs were re-stimulated for 24 h with or without antigen (Mtb sonicate). Cells cultured in medium alone served as control for cells cultured with antigen. RNA was isolated from these samples using miRNAeasy kit (Qiagen, Germantown, MD, USA) and then subjected to hybridization using miRNA 4.0 Affymetrix gene chips (Affymetrix, Santa Clara, CA, USA). The data was then subjected to statistical and bioinformatical analyses. A heat map of statistically significant (*p* ≤ 0.05; fold change ≤−2 or ≥2) miRNAs was generated and samples were clustered in a hierarchical fashion. Green color indicates a decrease in intensity, whereas red color indicates an increase; (**B**) Principal component analysis (PCA) plot depicts the clustering of RNA samples of 3 groups of rats, each tested in triplicates; (**C**) Venn diagram shows the distribution of statistically significant miRNAs whose expression was altered by antigen (Left-tilted panels) versus antigen-cum-drug (Right-tilted panels) under the indicated sub-groups.

The results of the miRNA expression of rats in the Incubation phase of AA and vehicle-treated arthritic rats are shown as a heat map (Figure 3A), principal component analysis (PCA) (Figure 3B), and Venn diagram (Figure 3C, left-titled panels). A comparison of the intensity signals of the two groups of rats revealed a total of 903 significantly altered “miRNA elements” in arthritic rats compared with Incubation phase rats (Figure 3C, left-titled panels). Of these, 748 were upregulated, which included 159 that were uniquely upregulated by antigen, meaning that celastrol treatment (described below) had no effect on these. The remaining 155 (of 903 elements) were downregulated in arthritic rats. These included 112 that were uniquely decreased by antigen, implying that these were unaffected by celastrol treatment.

**Figure 4:**
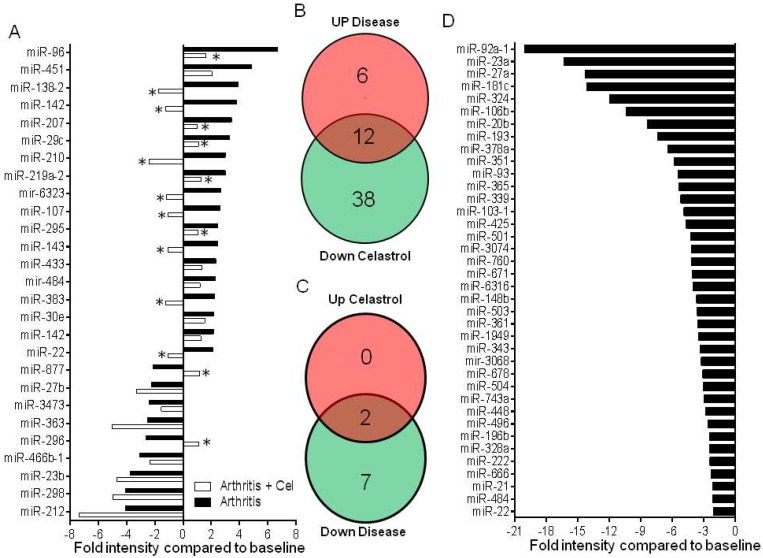
The differential expression of miRNAs in untreated arthritic rats versus celastrol-treated rats. (**A**) The miRNAs that are differentially expressed in arthritic rats compared to baseline control are shown in filled bars. The levels of all filled bars are statistically significant (*p* < 0.05) for at least one of 3 species’ probes (mouse, human, rat) in the microarray chip. The respective miRNAs in celastrol-treated rats are shown in open bars. Here, an asterisk (*) represents significant down- or upregulated miRNAs upon celastrol treatment compared to disease controls; (**B**,**C**) Venn diagrams showing the number of miRNAs that are up- or down-regulated in untreated arthritic rats compared with celastrol-treated rats; (**D**) The miRNAs that are uniquely downregulated in celastrol-treated group compared to the untreated group (meaning that they are not changed upon disease development) are shown here as a Waterfall plot.

Following extensive analysis using Ingenuity Pathway Analysis (IPA) and target prediction software, we examined in detail 27 miRNAs for further consideration (Figure 4A). Out of 27, 18 showed increased expression in disease (Figure 4B), whereas 9 had reduced expression in disease (Figure 4C).

### 2.2. LNC Micro-RNA Expression Profile of Celastrol-Treated Arthritic Rats

The results, including the heat map (Figure 3A), PCA (Figure 3B), and Venn diagram (Figure 3C, right-tilted panels), of the miRNA testing of LNCs of celastrol-treated rats are shown. A comparison of the celastrol-treated rats and the vehicle-treated arthritic rats revealed a total of 1336 differentially expressed miRNA elements (Figure 3C, right-tilted panels). Of these, 1231 were downregulated, while 105 were upregulated in celastrol-treated rats. Of the 1231 downregulated miRNA elements, 632 were uniquely downregulated by celastrol implying that these were not affected by antigen, whereas 12 were reduced by antigen and then further downregulated by celastrol. The remaining 587 were increased by antigen, but reduced by celastrol. On the contrary, of the 105 miRNA upregulated elements in celastrol-treated rats, 72 were uniquely increased by celastrol in that antigen had no significant effect on them, whereas 31 were decreased by antigen but increased by celastrol. The remaining 2 were increased by antigen and then further upregulated by celastrol.

The relative levels of expression of select miRNAs of arthritic rats were then compared with celastrol-treated rats (Figure 4). The miRNAs affected by celastrol treatment belonged to 3 different categories (Figure 4B,C): 12 increased in disease but reduced by celastrol; 38 reduced by celastrol, but unaffected by disease; and 2 decreased in disease but upregulated by celastrol. Figure 4D shows the relative expression levels of those 38 miRNAs reduced by celastrol but not affected by the disease. These 38 miRNAs might be reduced by the direct effect of celastrol, involving mechanisms not related to the disease condition. Among these is miR-22, which has two different miRNA types, such that Figure 4A shows miR-22-5p, whereas Figure 4D shows miR-22-3p.

### 2.3. Functional Analysis of Specific Micro-RNAs Identified Following Initial Screening

**Figure 5:**
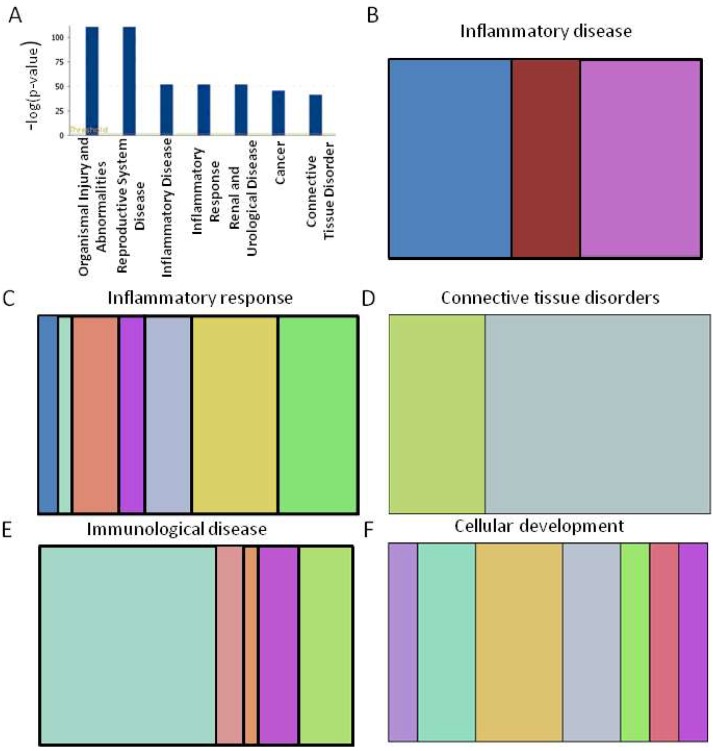
In silico analysis of miRNAs that are modulated following AA induction. (**A**) Bar graph indicating the number of miRNAs that are predicted to target genes in each category listed on the x-axis; (**B**–**F**) Vertical slice plots depict the percentage of miRNA-associated molecules and their indicated categories. The details of the distribution of miRNAs in different colored vertical slices and the categories of each plot are given in Supplementary Materials Figure S1A,B, and additional pathways in Supplementary Materials Figure S2A–C.

To examine the potential biological functions of the select miRNAs, the data was subjected to IPA Core analysis. The selected genes were then further grouped into functional categories and plotted as a bar graph (Figure 5A). This analysis revealed several different functional categories including inflammatory disease, inflammatory response, immunological disease, cellular development, connective tissue disorder, and others (Figure 5B–F). A detailed list of these categories is given in Supplementary Materials Figure S1A,B. Additional pathways are shown in Supplementary Materials Figure S2A–C. The above analyses indicated that these pathways are quite likely to be influenced by miRNAs induced upon arthritis development.

### 2.4. The Micro-RNA-Messenger RNA Interactions and Network Mapping

**Figure 6:**
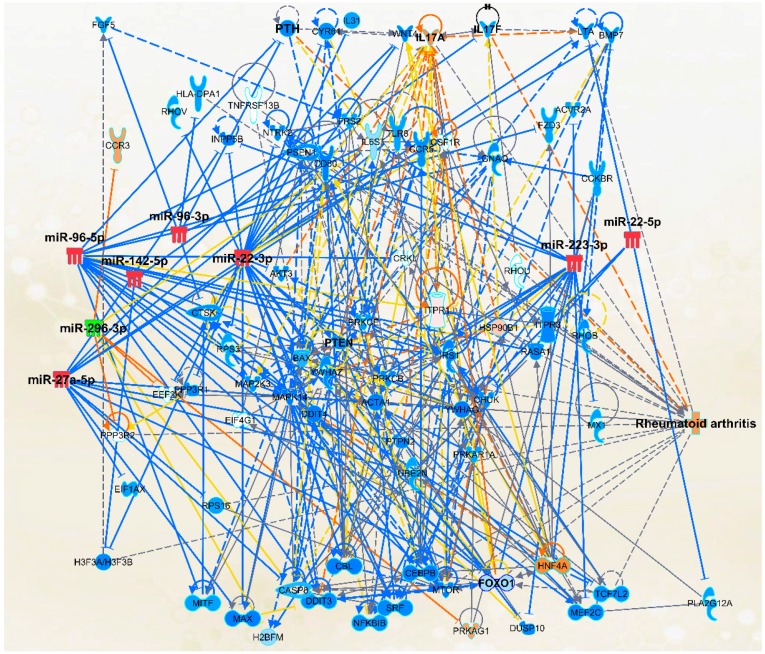
Network analysis of select miRNAs and the mRNAs targeted by them, as well as their impact on the progression of rheumatoid arthritis. The known interactions between the genes are represented by lines showing activation (arrow) or inhibition (blunt end). Further, solid line indicates direct interaction, whereas dashed line indicates indirect interaction. Colored lines indicate the following: orange line for activation; blue line for inhibition; yellow line for uncertain state of the downstream molecule; and gray line for effect not predicted. For the Micro-RNA symbols, red indicates increased level, whereas green indicates decreased level. For the target genes, orange indicates predicted activation, whereas blue indicates predicted inhibition.

**Figure 7:**
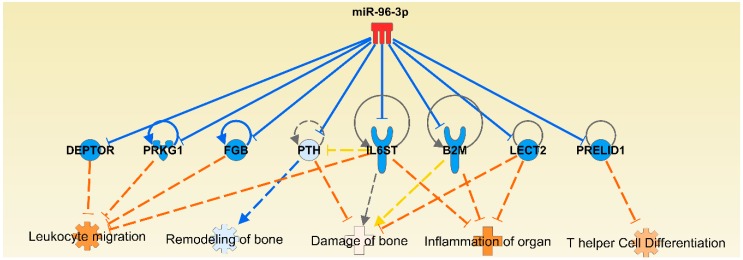
Network analysis showing the impact of miR-96 on various mediators and pathways involved in the pathogenesis of rheumatoid arthritis. The impact of an increase in the level of miR-96 on arthritis is also shown here. The description of lines, arrows, color, etc. is same as in the legend to Figure 6.

**Figure 8:**
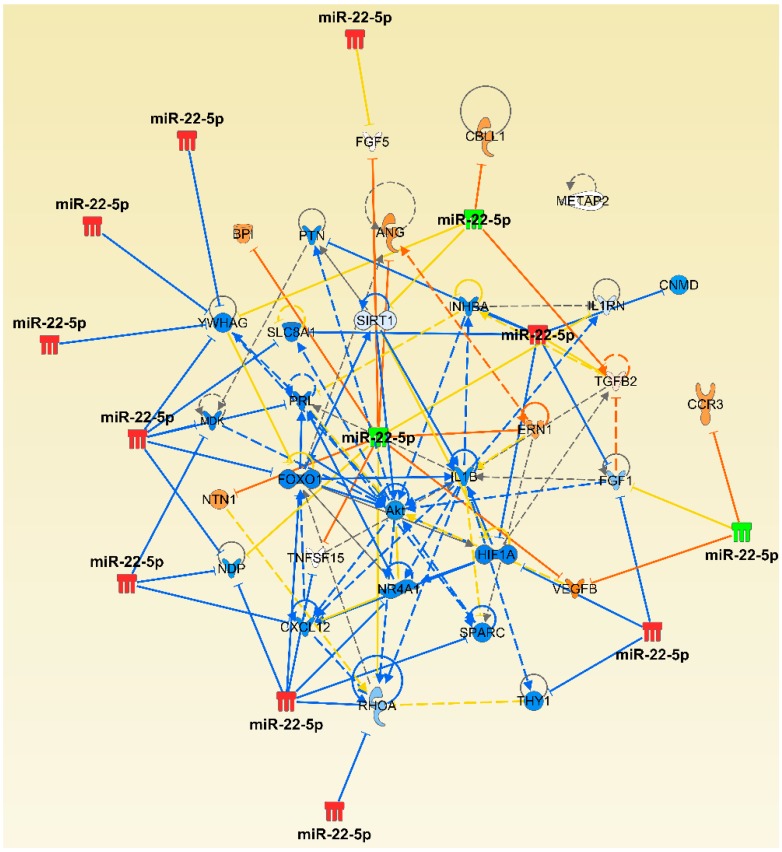
Network analysis of select miRNAs and the mRNAs targeted by them, as well as their impact on the proliferation of endothelial cells in inflammatory arthritis. The description of lines, arrows, color, etc. is same as in the legend to Figure 6.

We also examined the key miRNA-mRNA interactions using the network mapping tool (Figure 6, Figure 7 and Figure 8) for gaining further insight into how these miRNAs might affect different mediators and pathways in RA. The mediators that are associated with important pathways in RA were considered for further analysis. Interestingly, some of these pathways, such as IL-17 signaling and NF-κB signaling, which are known to be involved in RA, are also known to serve as targets of celastrol action [37,38].

### 2.5. Micro-RNA Expression in Endothelial Cells and Their Functional Relevance

Chronic inflammation, as in the case of human RA, is linked with endothelial dysfunction and aberrant new blood vessel formation (angiogenesis) [39,40,41]. In this regard, we also examined the involvement of miRNAs in endothelial activation and proliferation. Our network analysis (Figure 8) revealed that several miRNAs identified in lymphoid cells (e.g., LNCs) of arthritic rats modulate a variety of inflammatory mediators that affect endothelial cell activation/proliferation. Furthermore, some of these miRNAs are the same as those altered by celastrol in the LNCs described above. These results indicate that celastrol might also be able to modulate endothelial cell function via these miRNAs, in addition to its other anti-inflammatory effects as mentioned above.

### 2.6. Selection of Micro-RNAs as Biomarkers of Arthritis and Therapeutic Response

Thereafter, using multiple criteria, we selected 8 miRNAs, namely miR-22, miR-27a, miR-96, miR-142, miR-223, miR-296, miR-298, and miR-451, which have the potential to be key regulators of arthritis pathogenesis. Of these 8 miRNAs, five miRNAs (miR-22, miR-27a, miR-96, miR-142, and miR-223) were upregulated following disease development, but downregulated with celastrol treatment. However, two other miRNAs (miR-296 and miR-298) were reduced upon disease development. Of these, miR-296 was increased, but miR-298 was unaffected by celastrol treatment. Another miRNA (miR-451) was increased in disease, but not affected by celastrol treatment. We propose the above 8 miRNAs for further validation as biomarkers of disease activity in RA patients.

For the purpose of selecting biomarkers of therapeutic response, we preferred 6 of the 8 miRNAs, whose expression was changed significantly in disease and then further modulated by drug treatment. These include miR-22, miR-27a, miR-96, miR-142, miR-223, and miR-296. A representative network analysis of these is shown in Figure 6. Here, celastrol is used as a proof-of-concept drug. In this context, we propose that more than 6 miRNAs may serve as biomarkers of therapeutic response in RA patients. However, it is conceivable that another anti-arthritic drug might target a slightly different subset of miRNAs than celastrol. However, as our initial selection of miRNAs is based on the disease-related miRNAs, we anticipate that miRNAs targeted by celastrol would overlap with those affected by other anti-arthritis drugs.

### 2.7. Circulating Micro-RNAs in Sera of Rats

**Figure 9:**
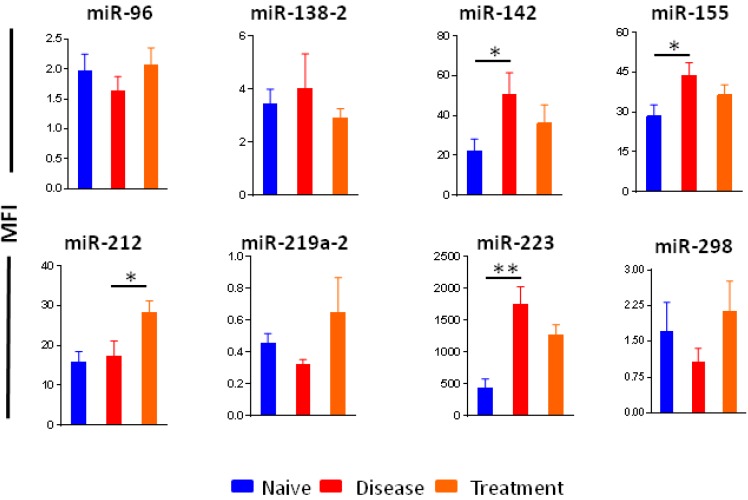
Testing miRNA levels in sera of rats. The levels of miRNAs were determined in serum samples obtained from normal (naïve) control, arthritic rats, and celastrol-treated arthritic rats (*n* = 6 each) using Multiplex miRNA assay. The data is presented as mean fluorescence intensity. (*, *p* < 0.05; **<0.025).

We then quantitated the levels of the above miRNAs, which were identified based on microarray miRNA expression analysis of LNC, in serum samples of control (naïve) rats, arthritic rats at peak phase of AA, and celastrol-treated rats at peak phase of AA. The levels of miR-142, miR-155, and miR-223 were higher in arthritic versus control rats, whereas miR-212 showed increased expression when comparing celastrol-treated and arthritic rats/ control rats (Figure 9). Changes in some other miRNAs had trends similar to the above 4 miRNAs, but the difference was not statistically significant. Another pattern observed with miR-96, miR-219a2, and miR-298 was a reduction in the level upon disease development, but reversal (increase) in level after celastrol treatment. However, the difference was not statistically significant.

### 2.8. Messenger RNA Targets of Select Micro-RNAs

Using multiple criteria, including the use of target prediction software, we identified the mRNA targets of the 8 selected miRNAs mentioned above. These targets are categorized into 3 groups (Table 1). We noted that our select miRNAs were highly predicted and/or experimentally observed to bind to the 3′ UTR of the mRNAs identified by us. For example, miR-96 is predicted to bind to mRNA encoding parathyroid hormone (PTH), which would result in reduced PTH activity (Figure 6 and Figure 7). PTH is an important hormone for mediating a homeostatic state of bone morphology, and can display anabolic or catabolic properties for bone remodeling depending on its concentration and pattern of secretion [42,43]. It has been shown that dysregulation of PTH can lead to an imbalance of critical mediators of bone remodeling. This in turn can lead to bone damage [43]. This situation can aggravate bone damage associated with inflammation of the joint in arthritis. Another interesting miRNA revealed in our analysis is miR-27a. This miRNA is predicted to target the gene Wingless-Type MMTV Integration Site Family, Member 4 (Wnt4) (Figure 6). While Wnt4 has also been associated with reproductive functions [44,45], it also plays a role in bone remodeling. For example, it has been demonstrated that Wnt4 can reduce the expression of NF-κB and subsequently inhibit bone resorption and inflammation [46]. This suggests that miR-27a could influence bone erosion and inflammation in part via Wnt4. Finally, miR-223 was predicted to target an important gene in RA, Forkhead Box O 1 (FOXO1) (Figure 6). FOXO1 has been shown to be reduced in expression in the peripheral blood of RA patients [47]. Furthermore, a reduction in FOXO1 promotes the survival of fibroblast-like synoviocytes (FLS). This implies that miRNA-induced changes in FOXO1 levels can influence FLS proliferation and joint inflammation. Our network analysis further revealed that when the expression of most of the above-mentioned miRNAs is decreased, for example, miR-96, the expression of their target mRNAs is increased, leading to the suppression of RA (Supplementary Materials Figure S3). The reverse effect is predicted when the expression of these miRNAs is increased.

## 3. Discussion

The pathogenesis of RA involves complex interactions among several mediators and pathways of inflammation and bone remodeling. Micro-RNAs are known to regulate a variety of mRNAs. Information about changes in miRNA expression following the development of arthritis can provide insights into disease pathogenesis, new biomarkers of disease, and potential therapeutic targets. In this context, we examined the miRNA expression profile of arthritic rats using the AA model. This experimental model of RA has extensively been used for decades to study the pathogenesis of human RA as well as several potential therapeutics [33,34]. However, at present, there is barely any information on the expression of miRNAs and their role in AA. Our study is the first in AA that provides comprehensive insights in this regard. Furthermore, we have used celastrol as a therapeutic agent to determine the changes in the miRNA profile of arthritic rats following treatment. Although several studies by others [48,49,50] and us [35,37] have uncovered the biochemical and immunological aspects of the anti-inflammatory properties of celastrol, the miRNA profile of arthritic rats following celastrol treatment has not been reported previously.

Here, we have combined the miRNA-microarray technology and bioinformatics-based analysis to comprehensively determine the miRNA expression profile of untreated and celastrol-treated arthritic rats. Analysis of these miRNA profiles uncovered a set of new potential biomarkers of arthritis progression as well as response to therapy. Our results show that 8 specific miRNAs (miR-22, miR-27a, miR-96, miR-142, miR-223, miR-296, miR-298, and miR-451) have the potential to be key regulators of arthritis pathogenesis. Of these, 6 miRNAs (miR-22, miR-27a, miR-96, miR-142, miR-223, and miR-296) are significantly modulated by celastrol treatment. Importantly, the significance of some of these miRNAs is further evident from the results of serum testing, which showed that miR-142, miR-155, and miR-223 were significantly higher in the sera of arthritic rats than normal rats. Furthermore, miR-212 showed increased expression when comparing arthritic rats and celastrol-treated rats. Changes in miR-96, miR-219a-2, and miR-298 also showed an interesting trend, reduction with disease but increase with celastrol treatment. We suggest that changes in miRNAs induced by celastrol treatment might be attributable to both direct effect of celastrol on miRNA expression and indirect effect of reduced inflammation. Taken together, the above miRNAs represent circulating biomarkers of disease activity as well as therapeutic response in AA. We believe that above-mentioned miRNAs, whether as a set or individually, could be used in conjunction with current biomarkers for improved diagnosis and/or prognosis of arthritis, as well as for monitoring responsiveness to therapeutic intervention.

We tested serum samples to determine which of the miRNAs that are found to be altered in immune cells (such as LNC) might also be changed in the blood (using serum). We believe that similar to serum RF and ACPA, a serum test to check for miRNA levels can be a useful adjunct for RA diagnosis. For that reason, we preferred serum over peripheral blood mononuclear cells (PBMC) so that in subsequent comparative studies, changes in serum miRNAs can be easily compared with changes in other biomarkers of RA. Regarding serum miRNAs, it is well documented that circulating miRNAs can be detected in many disease conditions [51,52,53,54]. Such miRNAs are produced by different cells/tissues, but some of these miRNAs are then released into circulation in the form of exosomes budding from living cells [51,52,53,54]. Another interesting function aspect of exosomes is that they can also be used as therapeutic targets or delivery systems [55,56,57,58]. In the above context, we hope that our results for serum miRNAs will be of practical utility for diagnosis and prognosis of arthritis.

A brief summary of the expression of specific miRNAs and their potential targets and effects in arthritis are given below:

miR-22. This miRNA was shown to be an important regulator of FLS. It has been demonstrated that miR-22 is able to directly target Cyr61, an important mediator that promotes FLS proliferation, and that miR-22 was downregulated in the synoviocytes of RA patients [59]. In turn, this led to an increase in Cyr61 expression and subsequent FLS proliferation. Moreover, in the collagen-induced arthritic (CIA) model of RA, miR-22 had an opposite effect. The inhibition of miR-22 resulted in increased suppression of proliferation and higher apoptosis rates of FLS, as well as a decrease in pro-inflammatory cytokine production [60]. Additional studies are required to resolve its action in arthritis.

miR-27a. There are a couple of reports describing the role miR-27a in RA. For example, miR-27a targets follistatin-like protein 1 (FSTL1), an important protein that promotes FLS migration and invasion [61]. In addition, it limits the TLR4/NF-kB pathway. In RA patients, miR-27a is downregulated in the serum, synovial tissue, and FLS, suggesting that it plays a crucial role in FLS proliferation and RA pathogenesis [61]. Another study revealed that miR-27a expression is reduced in chondrocytes in osteoarthritis, and that this miRNA downregulates, albeit indirectly, two critical genes involved in the pathogenesis of osteoarthritis, namely insulin-like growth factor binding protein-5 (IGFBP-5) and matrix metalloproteinase-13 (MMP-13) [62]. A recent study in a mouse model of arthritis showed that miR-27a controls arthritis via peroxisome proliferator activated receptor gamma (PPARγ), which also involved a similar set of genes as above [63]. Also, miR-27a levels were altered in RA patients following treatment with Adalimumab and/or Methotrexate [21], suggesting that this miRNA can be used to assess the patient’s responsiveness to therapy.

miR-96. To the best of our knowledge, there is no prior report yet on a direct association between arthritis and miR-96, and therefore, our study has revealed miR-96 as a promising candidate for further exploration. However, a few in vitro studies have pointed to some likely ways in which miR-96 might influence the disease process in arthritis. For example, the expression of miR-96 was found to be markedly reduced in chondrogenesis in mice when testing chondroblasts derived from mouse marrow stromal cells [64]. In another study, miR-96 was shown to facilitate osteogenic differentiation in MC3T3-E1 cells, a mouse osteoblast cell line [65]. This activity involved inhibition by miR-96 of heparin-binding EGF-like growth factor (HBEGF)-epidermal growth factor receptor (EGFR) signaling. Similarly, miR-96 was reported to regulate gene expression vital for human mesenchymal stromal cells (hMSCs) [66].

miR-142. This miRNA has been involved in signaling leading to enhanced inflammation. In regard to joint pathology, the expression level of miR-142 was found to be reduced in the cartilage in a mouse model of osteoarthritis [67]. Furthermore, over-expression of miR-451 resulted in inhibition of chondrocyte apoptosis and inflammation in these mice. This effect involved a reduction in high mobility group box 1 (HMGB1)-induced NF-κB signaling.

miR-155. Our results of serum testing showed that miR-155 is increased in arthritic rats. This observation is supported by a similar finding of increased miR-155 in sera and synovial tissue of RA patients [7,68,69]. This miRNA modulates several pro-inflammatory cytokines, chemokines, and chemokine receptors involved in arthritis pathogenesis. Furthermore, mice lacking miR-155 are resistant to CIA, further validating the role of this miRNA in arthritis [7].

miR-212. Another miRNA found to be increased in celastrol-treated rats compared with arthritic and control rats was miR-212. Thus, reduction in the severity of arthritis correlated with increase in miR-212 in serum. This finding is supported by the observation in RA sera and synovial tissue, where miR-212 is reduced in RA compared to healthy controls [70,71]. This miRNA modulates the proliferation and activity of FLS by targeting SRY-related HMG box 5 (SOX5) [71].

miR-223. It has been demonstrated that this miRNA is overexpressed in RA patients compared to healthy controls [69]. This overexpression led to a decrease in insulin growth factor-1 receptor (IGF-1R), subsequently impairing IL-10 activation in RA cells [72]. This impairment contributes to the imbalance of pro-inflammatory and anti-inflammatory cytokines, and exacerbates arthritis. Similarly, a study in a mouse model of arthritis revealed that silencing of miR-223 reduced the severity of the disease, indicating a role of this miRNA in disease progressions [73]. On the contrary, another study in RA showed increased expression of miR-223 in the synovial tissue of RA patients and its inhibitory effect on osteoclastogenesis [74]. Thus, both pro- and anti-inflammatory effects of miR-223 have been observed.

miR-296. It has been shown that TNF-α stimulation of bone marrow-derived macrophages increased the expression of miR-296, along with two other miRNAs in our list, namely miR-27a and miR-298 [25].

miR-298. As mentioned above, the expression of miR-298 has been reported to be increased following stimulation of macrophages with TNF-α [25]. While miR-298 currently has no reported role in RA, its expression has been shown to be increased in lupus patients compared to controls [75].

miR-451. As for miR-451, it has been shown to play a key role in controlling neutrophil chemotaxis, and is present in lower amounts in RA patient neutrophils compared to healthy controls [76]. This miRNA can directly target copine III (CPNE3) and Ras-related protein Rab-5A (Rab5a), thereby suppressing the p38/MAPK pathway. Furthermore, the over-expression of miR-451 in arthritic mice led to a reduction of neutrophil chemotaxis and severity of arthritis [76]. Similarly, in another study involving FLS from RA patients, it was shown that transfection of FLS with miR-451 resulted in inhibition of pro-inflammatory cytokines such as TNF-α, IL-1β, and IL-6, thereby supporting the anti-arthritic effect of this miRNA [77]. Furthermore, this effect of miR-451 involved inhibition of p38MAPK. On the contrary, another set of reports showed that miR-451 was increased in the RA sera [69] and T cells of RA patients, and that its levels correlated positively with serum IL-6 and arthritis severity [78].

In regard to arthritis therapy, with only about 60% of patients responding to the biologics [8], and the therapies taking weeks before clinical changes might be evident, there is a need for better biomarkers to monitor patient responsiveness. In this study on AA, we have shown that not only are miR-22, miR-27a, miR-96, miR-142, and miR-223 upregulated following disease development, but they are also downregulated with celastrol treatment. Furthermore, miR-296 was reduced upon disease development, but increased upon celastrol treatment. Upon serum testing, 3 miRNAs (miR-142, miR-155, and miR-223) were increased in arthritic rats, whereas one miRNA (miR-212) was increased in celastrol-treated rats compared with arthritic rats. Given that AA is a widely used model of RA and that miRNAs are highly conserved among species, our results suggest that above-mentioned miRNAs might serve as biomarkers to assess an RA patient’s response to therapy, and thereby indicate the need to switch to a different medication, if necessary. This in turn would help cut down on the time with therapies that are not efficacious at remedying disease morbidity. Moreover, based on our results, we further suggest that one or more of these 8 miRNAs might serve as effective targets for therapy. For example, a miRNA mimic can be delivered via a suitable vector to increase the expression of that miRNA in vivo with the purpose of suppressing the expression of a gene encoding a pro-inflammatory mediator of arthritis. The opposite can be achieved with an antagomir, an antagonist to the select miRNA by reducing the miRNA level but increasing the expression of the corresponding target mRNA for an anti-inflammatory cytokine or other regulatory protein.

Endothelial cell activation and proliferation, leading to the formation of new blood vessels (angiogenesis), is an integral part of the pathogenic events in arthritis [41,79,80]. Pro-inflammatory cytokines (e.g., TNF-α, IL-1β, IL-6, IL-17, and IL-18) and other inflammatory mediators (e.g., C-reactive protein) cause endothelial cell activation. Continuous endothelial cell activation enhances the expression of leukocyte adhesion molecules and chemokines, which in turn cause a sustained recruitment of leukocytes to the site of inflammation—as well as enhancing the levels of intracellular reactive oxygen species (ROS) and those of matrix metalloproteinases—and enhances angiogenesis [79,80]. These changes provide increased blood flow, nutrients, and inflammatory cells to the target site, the joints. Importantly, however, endothelial dysfunction has been reported not only in RA [39], but also in the rat AA model [40], further emphasizing the significant role of endothelial cells in arthritis. Therefore, a detailed study of the factors affecting endothelial cell activation/proliferation can provide insights into the disease process, and also offer vital clues to the molecules/pathways targeted by certain anti-arthritic agents that inhibit new blood vessel formation in arthritis [41]. As described above under Results, we observed that several miRNAs can modulate a variety of inflammatory mediators that affect endothelial cell activation/proliferation. Furthermore, celastrol can target some of the mediators and pathways involving these miRNAs. It is conceivable that these endothelial cell-related miRNAs can be targeted for developing novel therapies for rectifying the endothelial dysfunction observed in RA as well as for inhibiting new blood vessel formation in this disease. Taken together, we describe the changes above in miRNAs induced following celastrol treatment of arthritic rats. Previous studies in other disease models have similarly shown the effect of other natural products on different miRNAs, for example, that of resveratrol in colitis-associated tumorigenesis model [81].

## 4. Materials and Methods

### 4.1. Induction and Evaluation of Adjuvant-induced Arthritis (AA) in Lewis Rats

Animal experiments were performed following approval from the Institutional Animal Care and Use Committee (IACUC) of the University of Maryland School of Medicine, Baltimore (UMB), protocol# 0417011 (20 April 2017) and #0817006 (17 August 2017). AA was induced in a cohort of male Lewis rats (LEW/SsNHsd (RT.1^1^)) (Envigo, Madison, WI, USA), 5–6 weeks old, by subcutaneous injection at the base of the tail with heat-killed *M. tuberculosis* H_37_Ra (Mtb) (Becton, Dickinson and Company, Sparks, MD, USA) (1.5 mg/rat) suspended in mineral oil (Sigma-Aldrich, St. Louis, MO, USA). (Male rats were used for consistency with previous studies by others and us using the AA model.) Thereafter, the onset and progression of arthritis in the paws was evaluated daily or on alternate days. The grading of arthritis on a scale of 0 to 4 per paw was based on redness and swelling as described previously [35]. Furthermore, the total arthritic score for a rat is derived by the addition of the arthritic scores of all 4 paws. The onset of arthritis occurs about d 8–10 after Mtb injection, and the severity of AA peaks around d 18–19. The period between Mtb injection and onset of arthritis is the “Incubation phase”. The photographs, histological analysis, and computed tomographic (CT) imaging of hind paws was performed following procedures described elsewhere [35,37,82]. CT was performed at the Core for Translational Research in Imaging (C-TRIM), UMB.

### 4.2. Treatment of Arthritic Rats with Celastrol

Celastrol (Calbiochem, Darmstadt, Germany) was dissolved in phosphate-buffered saline (PBS) containing 0.1% dimethyl sulfoxide (DMSO) (Sigma-Aldrich, St. Louis, MO, USA) (PBS-DMSO). This celastrol solution (1.0 mg/kg) was then administered to rats via intraperitoneal (i.p.) injection beginning at the onset of the disease and then continued daily for 3 more days, followed by injection every other day until the day of euthanization. Control rats were injected with the vehicle (PBS-DMSO) on the corresponding days.

### 4.3. Lymph Node Cell (LNC) Culture and Total RNA Extraction

We harvested the draining LNCs from different groups of rats, cultured them in vitro, and isolated total RNA from them as described below.

#### 4.3.1. LNC of In Vivo Vehicle/Celastrol Treatment Group:

Arthritic Lewis rats, treated either with the vehicle (PBS-DMSO) or with celastrol in PBS-DMSO (=celastrol), were euthanized at the peak phase of AA (day 19 after Mtb injection) and their draining lymph nodes (superficial inguinal and popliteal) were collected. These lymph nodes were crushed between frosted glass slides (Fischer Scientific, Bridgewater, NJ, USA) to extract LNCs, which were then filtered through a mesh and washed. These LNCs were then cultured for 24 h at 37 °C in a six-well plate (5 × 10^6^ cells/well) in Dulbecco’s modified eagle medium (DMEM) containing 10% fetal bovine serum (FBS), 1% glutamine, 1% penicillin/streptomycin, and 0.1% β-mercaptoethanol, in the presence or absence of Mtb sonicate (10 µg/mL) as the antigen. (The latter was prepared by sonicating heat-killed Mtb described above and collecting the centrifuged supernatant.) The LNCs cultured in medium alone served as controls for cells cultured with antigen. The purpose of the in vitro re-stimulation of the LNC is to increase the level of expression of various immune mediators (e.g., miRNAs), and thereby to increase the sensitivity of their detection. This change involves an increase in the generation of total RNA, including miRNAs. In vitro restimulation of LNC with antigen is a standard protocol for testing various immune mediators during the course of a disease [35,37]. However, under the conditions of antigen restimulation in vitro, the naïve T cells or B cells will not be activated and not be able to skew the results of the assay. Therefore, we do not anticipate any negative effects of antigen restimulation on the measurement of miRNAs in this regard.

#### 4.3.2. LNC of Incubation Phase Rats:

LNCs of Mtb-immunized rats were harvested during the incubation phase of AA (day 5 after Mtb injection) and then cultured in the presence or absence of Mtb sonicate (10 µg/mL) as described above. The LNCs cultured in medium alone served as controls for cells cultured with antigen. Furthermore, the LNCs of Incubation phase rats served as the “baseline” controls for the above-mentioned LNCs of in vivo celastrol/vehicle treatment group.

#### 4.3.3. RNA Isolation from LNC of Rats:

Total RNA was extracted from LNCs of each of the above-mentioned groups of rats using miRNeasy mini kit (Qiagen, Germantown, MD, USA) following the manufacturer’s protocol. RNA concentration was determined spectrophotometrically using the NanoDrop ND-1000 (NanoDrop Technologies/Thermo Scientific, Wilmington, DE, USA), and the quality of RNA was determined by checking the ratio of 260/280 nm and 260/230 nm. RNA integrity number (RIN) was determined by the Bioanalyzer RNA 6000 Nano Kit (Agilent, Wilmington, NC, USA) following manufacturer’s protocol prior to using that RNA for miRNA-microarray analysis.

### 4.4. Micro-RNA Hybridization and Microarray Analysis

Following the schematic plan laid out in Figure 1, the expression profile of miRNAs in LNCs of the 3 groups of rats (celastrol-/vehicle-treated arthritic rats and Incubation phase rats), each in triplicate, was determined using GeneChip™ miRNA 4.0 Array (Affymetrix, Santa Clara, CA, USA). Raw microarray data (CEL files) were first pre-processed using the Robust Multi-Array Average (RMA) technique. The RMA algorithm was used to conduct background corrections, to normalize the distribution, and to summarize probe intensities. Thereafter, principal component analysis (PCA) was performed. The PCA covered 85.07% of the variability for the 3 groups of LNCs/rats. Following RMA and PCA, ANOVA was performed. After adjusting for *p*-value (<0.05) and more than two-fold change, those miRNA elements that met the cut-off values were identified. The microarray platform had miRNA probes of multiple species (e.g., rat, mouse, and human), as well as multiple probes of each species for a single miRNA. Therefore, the hybridization intensities for all such probes were collectively recorded as “miRNA elements”. In subsequent analysis, “miRNA” refers to a given RNA sequence represented by the probes of a single species.

We then employed Ingenuity Pathway Analysis (IPA) (Qiagen), as well as TargetScan, miRbase, and miRNA.org (available online: www.microRNA.org) software, to further analyze the top miRNAs and their potential gene targets. Using “Context score”, a score that determines how likely it is for a miRNA to bind to a specific gene target, the highest-scoring and most relevant genes for RA targeted by select miRNA were identified. We have also included in our analysis a few miRNAs (e.g., miR-27a) that are altered under in vitro settings (i.e., LNCs of arthritic rats treated with antigen/celastrol in vitro). This testing was necessary because the in vitro exposure of LNCs and other cell types to celastrol is required to carrying out some of the subsequent validation and mechanistic studies. In the Venn diagram depiction, “uniquely” up-/downregulated refers to change in miRNA elements that are evident in response to only one entity—antigen only or celastrol only—not by both. To examine the potential biological functions of the select miRNAs, the data was subjected to IPA Core analysis. The selected genes were then further grouped into functional categories and plotted as a bar graph. We also examined the key miRNA–mRNA interactions using the network mapping tool for gaining further insight into how these miRNAs might affect different mediators and pathways in RA.

### 4.5. Testing Micro-RNA Levels in Sera of Rats

The levels of miRNAs in sera of control (naïve) rats, arthritic rats at peak phase of AA, and celastrol-treated rats at peak phase of AA were tested using Multiplex miRNA assays with FirePlex Particle Technology (Abcam, Cambridge, MA, USA). Briefly, blood samples were collected from rats at the peak phase of arthritis or age- and sex-matched control rats. Thereafter, serum was prepared from blood and 20 μL of each sample was subjected to multiplex miRNA assay [83]. The results obtained by Multiplex assay were analyzed using FirePlex Analysis Workbench software. The data was presented after normalizing with three endogenous normalizers selected using the geNorm algorithm: miR-146a-5p, miR-451-5p and miR-17-5p.

## 5. Conclusions

In summary, this is the first study in the AA model to unravel 8 miRNAs (miR-22, miR-27a, miR-96, miR-142, miR-223, miR-296, miR-298, and miR-451) that can serve as biomarkers of disease. Of these, 6 miRNAs (miR-22, miR-27a, miR-96, miR-142, miR-223, and miR-296) can also serve as biomarkers of therapeutic response in arthritis to celastrol. At present, it is not clear if these 6 biomarkers would also be applicable to monitoring therapeutic response to other anti-arthritic drugs. We believe that this information would open new avenues for better diagnosis, prognosis, and treatment of autoimmune arthritis.

## Figures and Tables

**Table 1:** The target mRNAs of the selected top 8 miRNAs.

miRNA	High Prediction	Moderate Prediction	Experimentally Observed
miR-22	*CHUK*, *LTA*	*BCL2*, *CXCL13*, *IL36G*, *MMP1*, *PTK2*, *TGFA*, *VCAM1*, *TLR7*	*Cyr61*
miR-27a	*CASP8*, *CCR5*, *EIF1AX, GNAO1*, *INPP5B*, *RPS16, UBE2N*, *WNT4*	*BCL2*, *CD40LG*, *CD80*, *CXCL6*, *CXCR4*, *CXCR6*, *FLT1*, *TGFB1*, *WNT2*, *WNT6*, *LTA*, *FOXO1*, *IL10*, *IL1R1*, *IL2*, *IL2RA*	*FSTL1*
miR-96	*CTSB*, *CTSK*, *MTOR*, *PPP3R1*, *PRKAR1A*, *PRKCE*, *PTH*, *RARG*, *RASA1*, *TCF7L2*	*AHR*, *BCL10*, *BMP5*, *BMP8B*, *FGF9*, *FOXO3*, *FOXO4*, *HSP90AA1*, *IL17B*, *SMAD7*, *TGFBR1*, *TNFSF4*, *IL12A*, *IL22RA2*, *IL6ST*, *VEGFC*	*FOXO1*, *IRS1*, *MITF*
miR-142	*IL17F*	*FGF20*, *IL22*, *SOCS1*, *TNFSF13B*	*TGFBR1*
miR-223	*ACTA1*, *ACVR2A*, *CCKBR*, *DUSP10*, *FOXO1*, *HSP90B1*, *IL6ST*, *INPP5B*, *MX1*, *PTPN2*, *YWHAG*	*CCL11*, *CD86*, *DKK1*, *STAT1*, *FOXO3*, *IL23A*, *IL5*	*IRS1*, *RHOB*, *MEF2C*
miR-296	*CCR3*, *H2BFM*, *PPP3R2*, *PRKAG1*	*CD14*, *CD40LG*, *CXCL8*, *FGF1*, *ICAM1*, *IL6ST*, *MMP13*, *MMP8*, *VEGFB*	
miR-298	*CASP9*, *CHP1*, *EIF1*, *FKBP1A*, *IL1RN*, *RHOA*, *RHOG*, *VEGFB*, *WNT3A*	*SOCS3*, *TCF7L1*, *TLR9*, *ICOS*, *IL6R*, *VDR*, *CXCL13*, *CXCL5*, *CXCR3*, *DKK2*, *EGFR*, *MYD88*, *GATA3*, *IL10RA*, *IL12RB2*, *IL17F*, *IL2RB*	
miR-451	*ATF2*, *PSMB8*, *TSC1*	*BMP6*, *FGF5*, *IL6R*, *NFATC1*	*MIF*, *CPNE3*, *Rab5*

## Supplementary Materials

The following are available online at http://www.mdpi.com/1422-0067/19/8/2293/s1.

## Declaration

The contents do not represent the views of the U.S. Department of Veterans Affairs or the United States Government.
