# Supplementary material for: The Micro-RNA Expression Profiles of Autoimmune Arthritis Reveal Novel Biomarkers of the Disease and Therapeutic Response
Source: Int J Mol Sci. 2018 Aug 5;19(8):2293. doi: 10.3390/ijms19082293 (PMC6121685; doi:10.3390/ijms19082293)
Supplement: Supplementary file 1 [file ijms-19-02293-s001.pdf]

SUPPLEMENTARY MATERIAL

Figure S1A

B. Inflammatory disease

- 22.22%_Immunological Disease, Inflammatory Disease, Inflammatory Response, Neurological Disease, Organismal Injury and Abnormalities
- 38.89%_Connective Tissue Disorders, Inflammatory Disease, Inflammatory Response, Organismal Injury and Abnormalities, Respiratory Disease
- 38.89%_Inflammatory Disease, Inflammatory Response, Organismal Injury and Abnormalities, Renal and Urological Disease

C. Inflammatory response

- 14.58%_Connective Tissue Disorders, Inflammatory Disease, Inflammatory Response, Organismal Injury and Abnormalities, Respiratory Disease
- 14.58%_Inflammatory Disease, Inflammatory Response, Organismal Injury and Abnormalities, Renal and Urological Disease
- 25%_Inflammatory Response, Organismal Injury and Abnormalities
- 27.08%_Inflammatory Response
- 4.17%_Cell–To–Cell Signaling and Interaction, Cellular Function and Maintenance, Inflammatory Response
- 6.25%_Cell–To–Cell Signaling and Interaction, Cellular Function and Maintenance, Hematological System Development and Function, Inflammatory Response
- 8.33%_Immunological Disease, Inflammatory Disease, Inflammatory Response, Neurological Disease, Organismal Injury and Abnormalities

D. Connective tissue disorders

- 30%_Cancer, Connective Tissue Disorders, Organismal Injury and Abnormalities
- 70%_Connective Tissue Disorders, Inflammatory Disease, Inflammatory Response, Organismal Injury and Abnormalities, Respiratory Disease

Figure S1B

E. Immunological disease

- 13.04%_Immunological Disease
- 17.39%_Immunological Disease, Inflammatory Disease, Inflammatory Response, Neurological Disease, Organismal Injury and Abnormalities
- 4.35%_Hematological Disease, Immunological Disease
- 56.52%_Cancer, Hematological Disease, Immunological Disease, Organismal Injury and Abnormalities
- 8.7%_Cardiovascular Disease, DNA Replication, Recombination, and Repair, Developmental Disorder, Endocrine System Disorders, Hereditary Disorder, Immunological Disease, Neurological Disease, Organismal Injury and Abnormalities

F. Cellular development

- 18.18%_Cancer, Cellular Development, Cellular Growth and Proliferation, Organismal Injury and Abnormalities, Tumor Morphology
- 18.18%_Cellular Development, Cellular Growth and Proliferation
- 27.27%_Cellular Development
- 9.09%_Cancer, Cellular Development, Cellular Growth and Proliferation, Organismal Injury and Abnormalities, Respiratory Disease, Tumor Morphology
- 9.09%_Cellular Development, Cellular Growth and Proliferation, Connective Tissue Development and Function, Hematological System Development and Function, Hematopoiesis, Organismal Development, Tissue Development
- 9.09%_Cellular Development, Cellular Growth and Proliferation, Hematological System Development and Function, Hematopoiesis, Lymphoid Tissue Structure and Development, Tissue Development
- 9.09%_Cellular Development, Hematological System Development and Function

Figure S2A

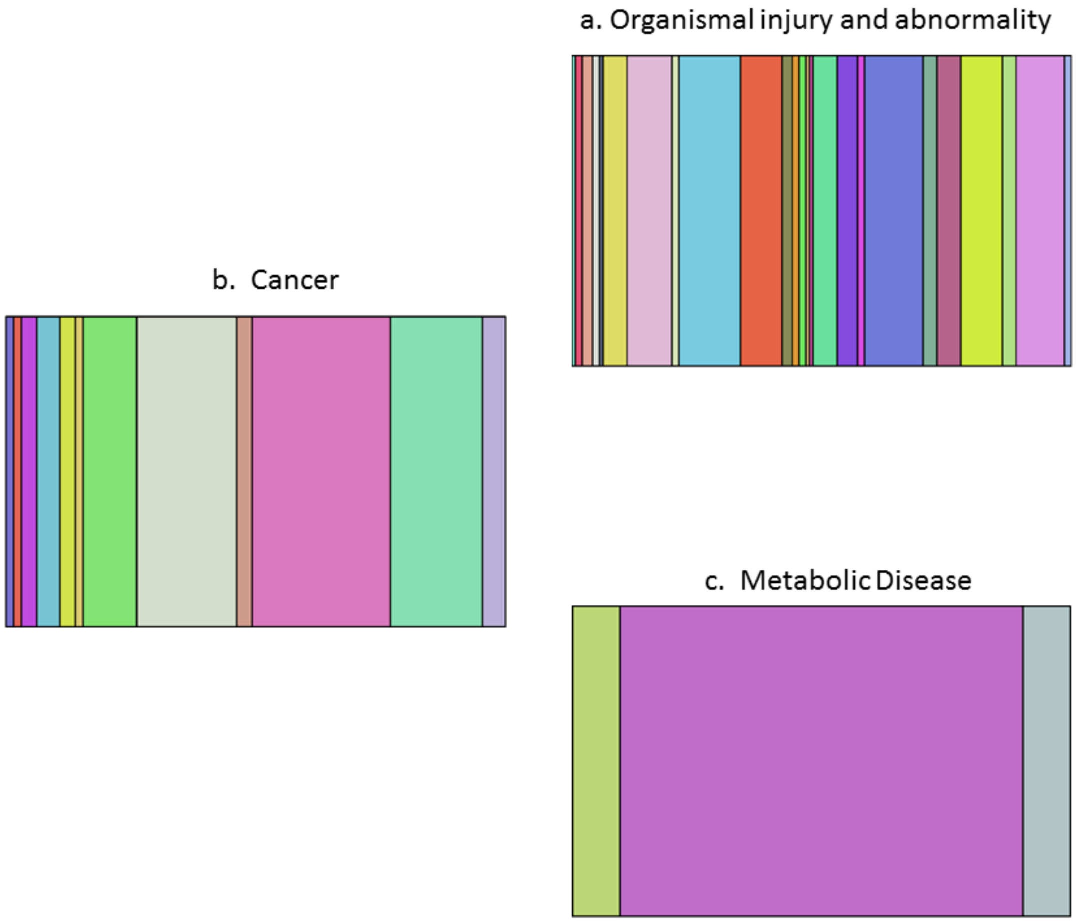
a. Organismal injury and abnormality
b. Cancer
c. Metabolic Disease

Figure S2B

a. Organismal injury and abnormality

- 0.69%_Cancer, Cellular Development, Cellular Growth and Proliferation, Organismal Injury and Abnormalities, Respiratory Disease, Tumor Morphology
- 0.69%_Cancer, Gastrointestinal Disease, Organismal Injury and Abnormalities
- 0.69%_Cardiovascular Disease, Organismal Injury and Abnormalities, Tissue Morphology
- 0.69%_Cellular Compromise, Organismal Injury and Abnormalities
- 1.38%_Cancer, Cellular Development, Cellular Growth and Proliferation, Organismal Injury and Abnormalities, Tumor Morphology
- 1.38%_Cancer, Endocrine System Disorders, Organismal Injury and Abnormalities
- 1.38%_Cancer, Neurological Disease, Organismal Injury and Abnormalities
- 1.38%_Cardiovascular Disease, DNA Replication, Recombination, and Repair, Developmental Disorder, Endocrine System Disorders, Hereditary Disorder, Immunological Disease, Neurological Disease, Organismal Injury and Abnormalities
- 1.38%_Cardiovascular Disease, Organismal Injury and Abnormalities
- 1.38%_Endocrine System Disorders, Gastrointestinal Disease, Hepatic System Disease, Metabolic Disease, Organismal Injury and Abnormalities
- 1.38%_Organismal Injury and Abnormalities, Skeletal and Muscular Disorders
- 11.72%_Endocrine System Disorders, Gastrointestinal Disease, Metabolic Disease, Organismal Injury and Abnormalities
- 12.41%_Cancer, Organismal Injury and Abnormalities
- 2.07%_Cancer, Connective Tissue Disorders, Organismal Injury and Abnormalities
- 2.07%_Cancer, Organismal Injury and Abnormalities, Reproductive System Disease, Skeletal and Muscular Disorders
- 2.76%_Immunological Disease, Inflammatory Disease, Inflammatory Response, Neurological Disease, Organismal Injury and Abnormalities
- 2.76%_Organismal Injury and Abnormalities
- 4.14%_Dermatological Diseases and Conditions, Organismal Injury and Abnormalities
- 4.83%_Cancer, Gastrointestinal Disease, Organismal Injury and Abnormalities, Respiratory Disease
- 4.83%_Connective Tissue Disorders, Inflammatory Disease, Inflammatory Response, Organismal Injury and Abnormalities, Respiratory Disease
- 4.83%_Inflammatory Disease, Inflammatory Response, Organismal Injury and Abnormalities, Renal and Urological Disease
- 8.28%_Cancer, Organismal Injury and Abnormalities, Reproductive System Disease
- 8.28%_Inflammatory Response, Organismal Injury and Abnormalities
- 8.97%_Cancer, Hematological Disease, Immunological Disease, Organismal Injury and Abnormalities
- 9.66%_Organismal Injury and Abnormalities, Reproductive System Disease

Figure S2C

b. Cancer

- 1.54%_Cancer
- 1.54%_Cancer, Cellular Development, Cellular Growth and Proliferation, Organismal Injury and Abnormalities, Respiratory Disease, Tumor Morphology
- 1.54%_Cancer, Gastrointestinal Disease, Organismal Injury and Abnormalities
- 10.77%_Cancer, Gastrointestinal Disease, Organismal Injury and Abnormalities, Respiratory Disease
- 18.46%_Cancer, Organismal Injury and Abnormalities, Reproductive System Disease
- 20%_Cancer, Hematological Disease, Immunological Disease, Organismal Injury and Abnormalities
- 27.69%_Cancer, Organismal Injury and Abnormalities
- 3.08%_Cancer, Cellular Development, Cellular Growth and Proliferation, Organismal Injury and Abnormalities, Tumor Morphology
- 3.08%_Cancer, Endocrine System Disorders, Organismal Injury and Abnormalities
- 3.08%_Cancer, Neurological Disease, Organismal Injury and Abnormalities
- 4.62%_Cancer, Connective Tissue Disorders, Organismal Injury and Abnormalities
- 4.62%_Cancer, Organismal Injury and Abnormalities, Reproductive System Disease, Skeletal and Muscular Disorders

c. Metabolic Disease

- 80.95%_Endocrine System Disorders, Gastrointestinal Disease, Metabolic Disease, Organismal Injury and Abnormalities
- 9.52%_Endocrine System Disorders, Gastrointestinal Disease, Hepatic System Disease, Metabolic Disease, Organismal Injury and Abnormalities
- 9.52%_Endocrine System Disorders, Hematological Disease, Metabolic Disease

Figure S3

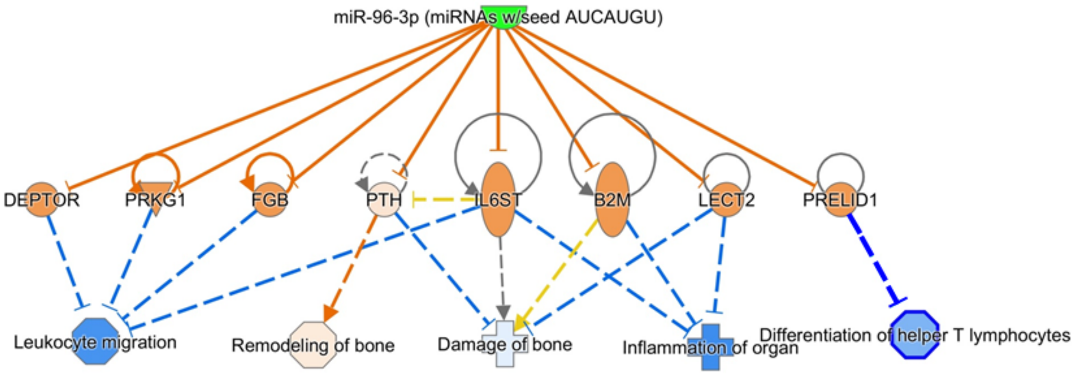
miR-96-3p (miRNAs w/seed AUCAUGU)
DEPTOR
PRKG1
FGB
PTH
IL6ST
B2M
LECT2
PRELID1
Leukocyte migration
Remodeling of bone
Damage of bone
Inflammation of organ
Differentiation of helper T lymphocytes
